# Inappropriate Shock Delivered By Implantable Cardioverter Defibrillator - Cardiac Resynchronization Therapy (ICD-CRT) Due To Myopotential Oversensing

**Published:** 2009-01-07

**Authors:** Hamid Barakpour, Zahra Emkanjoo, Abolfath Alizadeh, Mohammad Ali Sadr-Ameli

**Affiliations:** Department of Pacemaker and Electrophysiology, Rajaie Cardiovascular Medical and Research Center, Tehran, Iran

**Keywords:** Implantable defibrillator, myopotential oversensing, Inappropriate shock

## Abstract

The clinical efficacy of ICD-CRT therapy depends on accurate sensing of intracardiac signals and sensing algorithms. We report the occurrence of sensing abnormality in a patient with ICD-CRT. In this patient, oversensing of myopotentials during strenuous muscular activity resulted in an inappropriate ICD-CRT discharge. Although modern ICDs are highly effective in detecting and terminating malignant tachyarrhythmias, their detection specificity must be improved. It is possible to find the mechanism of arrhythmia by EGM. Simple device reprogramming make it possible to avoid the oversensing of myopotentials.

## Introduction

Inappropriate therapy (IT) for rhythm other than ventricular fibrillation (VF) and sustained ventricular tachycardia (VT) is the most common adverse event associated with implantable cardioverter defibrillators (ICDs). It occurs in 14-29% of patients, accounting for up to 50% of the total complications [1,2]. Most of the ITs are due to supraventricular tachycardia (SVT), such as paroxysmal supraventricular tachycardia (PSVT), atrial fibrillation (AF), and sinus tachycardia [3]. Other mechanisms are reconfirmation error due to a premature ventricular complex following a non-sustained ventricular tachycardia (NSVT), myopotential oversensing, T wave oversensing, lead fracture, device malfunction, electromagnetic interference, and far-field R wave oversensing [4,5].

This is the report of a rare case that myopotential oversensing was the cause of inappropriate therapy by an intact lead system of ICD-CRT.

## Case Presentation

The patient was a 50 - year-old male with a history of  coronary artery disease and previous myocardial infarction, with severe depression of left ventricular function and NYHA functional class III, who suffered from recurrent symptomatic ventricular tachycardia (VT). He had an ICD-CRT (Medtronic, InSync Marquis, model # 7277) implanted 3 years ago. A Medtronic lead (model # 6944) was placed in the right ventricular apex, another one (model # 5076) in the right atrium and the third one (model # 4193) in the lateral branch of the coronary sinus. ICD detection and treatment were programmed for three zones, ventricular fibrillation (VF) zone [320 ms, number of intervals detected (NID 9/12)], fast VT zone (via VF, 290 ms), VT zone (400 ms, NID 12/24). Antitachycardia pacing was programmed on. The sensed R wave amplitude was measured at 14 mV and the pacing threshold was 0.3 V and 0.9 V at 0.5 ms in the RV and the LV channels, respectively. During 3 years follow up after ICD implantation, the patient was followed regularly and had no episode of ventricular tachyarrhythmias or any discharge. No change in leads impedance was measured at different positions and no fracture or dislodgement was observed on chest radiographs.

The patient presented to the pacemaker clinic, complaining of a single shock without any warning symptom. The patient received the shock while throwing a metallic object forcefully with strong muscular contraction. No further shock was felt subsequently.

**Figure 1:**
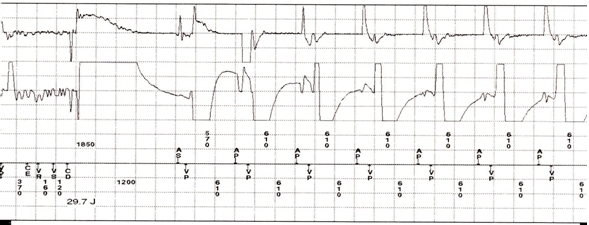


**Figure 2:**
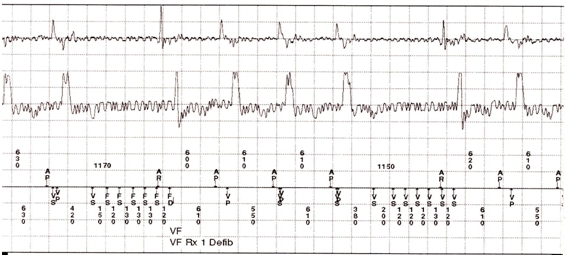
Intracardiac EGMs showing AV nodal jump followed by the initiation of slow-fast AVNRT.

Twenty days later, the patient presented for regular follow up at the ICD clinic. Upon interrogation of the device, the counters revealed one episode of VF, for which a 29.7 J shock was delivered at the time of mentioned event (Figure 1). EGM revealed regular appearance of R-waves at an interval of about 610 ms; however there was undulating high frequency noise distorting the baseline corresponding to the time of forceful muscular contractions (Figure 2). This noise disappeared after delivery of the shock. The device interpreted (detect) this episode as VF, resulting in capacitor charging for VF therapy (charge). To determine the mechanism of electrical signal oversensing, we replicated brief noise by asking the patient to make a sudden strong forward movement against resistance. Impedance of the high voltage lead was within the normal range (50 Ohm) and the episode lasted for 9 seconds. Further interrogation of the defibrillator did not reveal any detrimental alteration in the pacing/ICD leads or the ICD circuit. To avoid inappropriate shock, we programmed longer duration of detection in the VF zone. No recurrence was observed during follow-up with the newly programmed parameters.

## Discussion

This is a typical example of myopotential oversensing. Inappropriate therapy for rhythm other than ventricular fibrillation (VF) and sustained ventricular tachycardia (VT) is the most common adverse event associated with ICD implantation [6,7]. In the absence of damage to leads, electrical interference with ICD devices has rarely been identified as a cause of inappropriate therapy [8]. There are some reports about myopotential and / or diaphragmatic oversensing in pacemaker [9-13]. This is the report of a rare case with ICD-CRT  and intact lead system. Babuty et al reported inappropriate ICD discharge secondary to sensed myopotentials during deep breathing or the Valsalva maneuver and other situations without lead failure [12]. Oversensing of diaphragmatic myopotentials was primarily observed in patients implanted with defibrillator leads providing "integrated bipolar" sensing [14,15] In experience of Schulte et al  in 90% of cases the reduction of maximum sensitivity was effective in preventing further episodes of nonadequate arrhythmia detection [14]. Therefore by recording intracardiac electrogram it was possible to demonstrate that the mechanism was an oversensing of the activity of the pectoralis muscles, because the small amplitude of the muscular potentials was detected by the ICD device as VF. The problem can be solved by changing the sensitivity of the device or prolonging the time of detection of ventricular fibrillation to avoid inappropriate shock.
